# The Role of Phosphorus Sources and Phytase in Growth Performance and Feed Digestibility in Broilers

**DOI:** 10.3390/ani15142111

**Published:** 2025-07-17

**Authors:** Edouard Coudert, Nereida L. Corrales, Amélie Juanchich, Margot Poujol, Benjamin Ribeiro, Tristan Chalvon-Demersay, Guillermo Fondevila

**Affiliations:** 1 Centre Mondial de l’Innovation Roullier, 18 Avenue Franklin Roosevelt, 35400 Saint-Malo, France; 2 Departamento Produccion Agraria, Universidad Politecnica de Madrid, Moncloa-Aravaca, 28040 Madrid, Spain; 3 Phosphea, 27 Avenue Franklin Roosevelt, 35400 Saint-Malo, France; 4 Departamento Produccion Animal, Universidad Zaragoza, 12 C. de Pedro Cerbuna, 50009 Zaragoza, Spain

**Keywords:** phosphate, phytase, digestibility, broiler, nutrition optimization

## Abstract

Phosphorus (P) is an essential mineral in poultry nutrition, but its low bioavailability from conventional feed ingredients presents a significant challenge for efficient utilization. This study investigated the effects of different P sources (monocalcium phosphate (MCP) and calcium humophosphate (CHP)) and three phytase concentrations (0, 1000, or 2000 FTU/kg of feed) on broiler performance and ileal nutrient digestibility. A total of 600 Cobb 500 male broilers were fed one of six different dietary treatments with 10 replicates per treatment from 11 to 21 d of age. The birds and feed were weighed at 1, 10, and 21 d of age to determine growth performance. At 21 d of age, ileal digesta was collected from all the birds to determine dry matter, organic matter, Ca, P, and ash digestibility. The results indicated that phytase supplementation at 1000 and 2000 FTU/kg improved the feed conversion ratio and P digestibility compared with the non-supplemented group. The broilers fed CHP showed superior P digestibility compared to those fed MCP, regardless of phytase concentration. The results also indicated interactive effects between P source and phytase concentration on the digestibility of dry matter, organic matter, and ash. The findings suggest that CHP enhances nutrient digestibility and could optimize P utilization in broiler diets with phytase inclusion. This report reveals a better understanding of the effects of P sources and phytase concentration on feed digestibility. Further studies are needed to confirm these findings and support improvements in broiler performance under commercial production conditions.

## Simple Summary

Phosphorus is a vital nutrient in broiler nutrition, playing a key role in growth performance and skeletal development. However, its availability from plant-based feed ingredients is often limited, leading to challenges in meeting birds’ nutritional needs efficiently. Feed formulations commonly include inorganic phosphorus sources and enzymes, like phytase, to improve phosphorus utilization. Understanding how different phosphorus sources and phytase levels influence nutrient digestibility is essential to optimize feed efficiency and reduce environmental impact. This study explores the combined effects of dietary phosphorus source and phytase supplementation on broiler performance, offering insights to support more effective and sustainable feeding strategies.

## 1. Introduction

The poultry production sector plays a critical role in global food security, providing affordable and high-quality protein to a growing population. Optimal nutrition is essential not only for maximizing animal health and performance, but also for ensuring economic efficiency and sustainability across the value chain [1]. Phosphorus (P) is a vital mineral in poultry nutrition, playing a crucial role in key physiological processes such as bone development, energy metabolism, and nucleic acid synthesis. However, the efficient utilization of P in broiler diets remains a critical concern due to its low bioavailability in conventional plant-based feed ingredients [2]. To address this challenge, the inclusion of alternative feed components has become increasingly important, and novel inorganic or organic P sources with higher bioavailability appear as promising candidates. In addition, exogenous phytases have emerged as a promising strategy to enhance P utilization by hydrolyzing phytic acid, a major anti-nutritional factor present in plant-based feedstuffs. Numerous studies have underscored the significance of phytase supplementation in improving phosphorus availability in broiler diets [3]. However, limited research has systematically compared the efficacy of different P sources fortified with varying phytase concentrations, especially with a focus on the ileal digestibility of minerals, dry matter (DM), and organic matter (OM).

Humic substances, complex organic compounds derived from decomposed OM in soil, have recently garnered attention for their potential to modulate mineral metabolism in poultry. While studies have explored the impact of humic substances on nutrient utilization in livestock, a comprehensive examination of their effects on mineral digestibility and efficiency in broilers remains a compelling area of investigation. Several studies have highlighted the positive effects of humic substances on nutrient utilization and gut health in broilers [4]. Nevertheless, limited research has investigated the specific impact of humic substances on mineral digestibility and efficiency in broilers.

Mineral availability is often hindered by anti-nutritional factors present in feed ingredients, necessitating the exploration of innovative strategies to enhance mineral utilization. Humic substances have been proposed as potential candidates for improving mineral absorption by promoting favorable changes in gut physiology and microbial activity in other livestock species, like dairy cows and weaning piglets [5]. However, a thorough understanding of the nuanced interactions between humic substances and mineral digestion in broilers is essential to harness their full potential in commercial poultry production.

Our laboratory designed a specific process to generate a chemical reaction between a calcium source, humic substances, and a purified phosphoric acid, leading to a new raw material synergizing humic substances and P source effects, called calcium humophosphate (CHP). This CHP aims to have a double mode of action in enhancing P utilization in broiler nutrition: greater bioavailability of P, and a mitigating effect against anti-nutritional factors in feed. This research compares two phosphorus sources—monocalcium phosphate (MCP) and CHP—at varying phytase concentrations to evaluate their effects on broiler ileal digestibility at 21 d of age. MCP is commonly used on commercial farms as a basic phosphorous source to fulfill animal requirements. These findings aim to guide the formulation of nutritionally balanced diets, ultimately enhancing the health and performance of broiler chickens in commercial conditions. Our investigation aims to better understand the interplay between P sources, phytase concentrations, and nutrient digestibility in broiler nutrition in order to optimize nutrient utilization in commercial poultry production. Our study is based on the hypothesis that the P sources and phytase concentrations in the feed interact with each other, having an impact on nutrient digestibility and the overall performance of broilers.

## 2. Materials and Methods

### 2.1. Ethical Committee

The procedures used in this research were approved by the Animal Ethics Committee of Universidad Politécnica de Madrid (protocol reference: EOTUOHAAMP-GGM-ANIMALES-20230125) and were in compliance with the Spanish Guidelines for the Care and Use of Animals in Research (Boletín Oficial del Estado, 2013).

### 2.2. Experimental Ingredients and Diets

Two phosphate sources were evaluated in this research: MCP and CHP. Both P sources were analyzed for ash, calcium (Ca), and P prior to feed formulation. In addition, the Ca content of the experimental batch of limestone was determined. The chemical values used in feed formulation for these ingredients were as follows: (a) 81.1% ash, 18.0% Ca, and 22.4% P for MCP; (b) 78.2% ash, 16.0% Ca, and 21.4% P for CHP; and (c) 98% ash and 37.8% Ca for limestone. The feeding program consisted of 2 periods: starter (from 1 to 10 d of age) and grower (from 11 to 21 d of age). A common crumbled diet was fed during the starter period. The starter diet was formulated to contain 2950 kcal AMEn/kg, 21.9% crude protein, 1.22% digestible lysine, 0.94% Ca (FEDNA, Spain, 2021), and 0.58% total P, and was supplemented with 1000 FTU Axtra^®^ PHY per kg of feed. The phytase source was added during the feed production process via premix. For the grower period (11 to 21 d of age), two basal diets free of phytase were formulated to contain 3030 kcal AMEn/kg, 20% crude protein, 1.08% digestible lysine, 0.51% total P, and 0.72% Ca (FEDNA, 2021), including MCP or HMP as the unique source of phosphate. The ingredient composition and chemical analysis used for validation of experimental diets are available in Table 1. The analyzed chemical composition of the phosphate sources and limestone under test was used in feed formulation. The phytate phosphorus and phytic acid concentrations of the basal diets were calculated as 0.26% and 0.92% of the feed, respectively [6]. Six experimental diets were defined based on the phosphate source (MCP vs. CHP) used in the basal diet and three levels of phytase supplementation (0, 1000, or 2000 FTU/kg of feed) in the grower diet. The phytase inclusion rates are based on current commercial practices and discussions with feed formulators. All the grower diets were fed in pellets and included 0.5% of TiO_2_ as an indigestible marker for further digestibility determinations. The calculated chemical compositions of the experimental diets were confirmed by laboratory analysis.

### 2.3. Bird Husbandry

A total of 600 Cobb 500 male broilers were received from a commercial hatchery at 1 d of age. Chicks were weighed and randomly distributed in groups of 10 birds into 60 cages. From 1 to 10 d of age (starter period), all birds received a common diet, and then, from 11 to 21 d of age (grower period), each cage received the corresponding experimental diet according to treatment (10 cages per treatment). The cages (1.45 × 0.45 m^2^) were equipped with two low pressure nipple drinkers and an open through feeder with an adjustable door. In addition, first-age feeders and drinkers were used for the first week of age. All birds had free access to feed and water throughout the experiment. Environmental conditions were controlled automatically and adapted to commercial practices throughout the experiment. Room temperature was maintained at 33 °C during the first 2 d of age, and then it was reduced gradually until reaching 25 °C at 21 d of age. Chicks received a light program of 24 h light for the first 2 d of age, and then the number of hours of light per day was reduced by 1 h/d until reaching 18 h light at 7 d of age. During the trial, animal mortality did not exceed 1%.

### 2.4. Growth Performance

Body weight and feed ingestion were determined by cage at 10 and 21 d of age. Bird mortality was recorded and weighed as produced and used to correct feed conversion ratio (FCR) values. From these data, the average daily gain (ADG), average daily feed intake (ADFI), and FCR were determined by period (1 to 10 d and 11 to 21 d of age) and cumulatively (1 to 21 d of age).

### 2.5. Ileal Digestibility of Nutrients

At 21 d of age, all chicks were euthanized by CO_2_ asphyxiation and the ileal contents were collected from the terminal third part of the ileum. The ileum was defined as the portion of the small intestine extending from Meckel’s diverticulum to 2 cm cranial to the ileo-cecal junction. The ileal digesta was collected by gently flushing with distilled water into plastic containers, pooled by cage, and immediately frozen at −80 °C with dry ice. Then, the samples were stored at −20 °C until further determination of Ca, P, ash, and titanium (Ti) contents. From these data, the ileal digestibility of DM, OM, Ca, P, and ash were calculated according to the following expression:

Ileal digestibility: X (%) = 100 − [(Ti_diet_ × X_ileal_)/(Ti_ileal_ × X_diet_)] × 100

in which X was the nutrient considered for the calculations and the chemical compositions of the diets that were used. The OM was calculated using the following formula: Dry Matter − (minus) Ash.

### 2.6. Statistical Analysis

Growth performance and ileal nutrient digestibility data were analyzed using the GLM procedure in SAS 9.4M7 (SAS Institute Inc., Cary, NC, USA) based on a 2 × 3 factorial arrangement within a randomized complete block design. This design accounted for the main effects and their interactions. The cage, housing 10 birds, was considered the experimental unit for all measurements. Statistical significance was established at *p* < 0.05, with trends toward significance noted at *p* < 0.10. Pairwise comparisons among means were conducted using Tukey’s *t*-test.

## 3. Results

### 3.1. Growth Performances

The effects of the phosphate source and the phytase activity of the grower diet on body weight and growth performance of the birds from 0 to 21 d of age are presented in Table 2. No interactions between main effects were detected for any of the experimental periods considered. As expected, no differences among the treatments were detected from 1 to 10 d of age, a period in which all the birds received a common starter diet. From 11 to 21 d of age, the source of phosphate did not affect the ADG, ADFI, or FCR.

However, the FCR was improved in the birds fed with 1000 or 2000 FTU/kg compared to those fed with diets free of phytase (*p* < 0.01), an effect that was also observed for the cumulative period from 1 to 21 d of age (*p* < 0.05).

### 3.2. Ileal Digestibility

The effects of the source of phosphate and the phytase activity of the grower diet on nutrient digestibility at 21 d of age are presented in Table 3. The statistical analyses showed a significant effect of phytase dose (*p* = 0.001) and phosphorus source (*p* = 0.001) on ileal phosphorus digestibility in the chickens. However, no significant interaction between these two factors was observed (*p* = 0.271), indicating that their respective effects are independent.

As no interactions between the main effects were detected for P digestibility, significantly greater values were observed in the broilers fed CHP than in those fed MCP at all doses of phytase (*p* < 0.001). In addition, the P digestibility increased from 0 to 2000 FTU/kg (*p* < 0.001). Furthermore, the inclusion of CHP in the substitution of MCP as the unique source of mineral phosphate in the diet improved the ileal digestibility of DM, OM (*p*< 0.001), and ash (*p* = 0.01) with phytase interaction. The effects were greater at 0 and 1000 FTU/kg (*p* < 0.01) than at 2000 FTU/kg of phytase inclusion (*p* < 0.05 for the interaction between main effects).

## 4. Discussion

### 4.1. Growth Performances

Table 2 shows the effects of phosphate source and phytase activity in the grower diet on broiler growth from 1 to 21 d. No differences were observed from 1 to 10 days, as all the birds received the same starter diet. From 11 to 21 d, the inclusion of CHP in the substitution of MCP in the diet had no impact on ADG, ADFI, or FCR. In this respect, Varella et al. (2025) reported improved growth performance in broilers fed CHP compared with those fed MCP from 1 to 21 d of age [7]. However, the results reported herein suggest that during this rapid growth phase of young broilers, in which the digestive efficiency of the gastrointestinal tract is limited, [8], small differences in phosphorus availability may not significantly affect bird performance in all cases. Both phosphate sources could be considered as bioavailable sources of P compared to others, such as dicalcium phosphate [9].

The broilers fed 1000 or 2000 FTU/kg phytase showed improved FCR compared to those fed phytase-free diets (*p* < 0.01) from 11 to 21 d of age, with a similar effect observed over the entire 1–21 d period (*p* < 0.05). These results agree with previous research indicating significant improvements in broiler performance by phytase supplementation in the diet [10,11,12]. Diets containing corn, soybean meals, and/or wheat bran contain significant amounts of phytates (up to 2%). Phytate concentrations exceeding 0.25% in the diet might produce an antinutritional effect, significantly decreasing ADG and minerals’ absorption (P, Ca, Zinc) [13]. The beneficial effect on FCR in response to phytase supplementation in the diet may be associated with an increase in phytate hydrolysis. In this respect, the addition of phytase has a direct impact on phytate (IP6) concentration, but also on the phytate ester (inositol—IP5, IP4 and IP3) concentrations in the digestive tract, leading to better feed efficiency, health parameters, and mineral utilization (bone ash, plasma dosages) [14].

### 4.2. Ileal Digestibility

Table 3 shows the effects of phosphate source and phytase dose on nutrient digestibility at 21 d. Both phytase dose and phosphate source significantly affected ileal P digestibility (*p* = 0.001), with no interaction between the two factors (*p* = 0.271), indicating independent effects. In this respect, phosphorus digestibility was higher with CHP than with MCP across all the phytase levels and, as expected, increased with phytase dose. Additionally, dietary CHP improved DM, OM, and ash digestibility, a significant effect that was especially evident at 0 and 1000 FTU/kg. These data suggest that the benefits of the inclusion of CHP in the diet might apply beyond enhancing phytase efficacy. The improvement in OM digestibility may be due to a reduction in the antinutritional effect of phytate, resulting in a reduction in Ca-induced complexes that impair the availability of nutrients such as proteins and/or amino acids [15]. The CHP, as a new chemical complex, has been shown to improve mineral digestibility and availability in broiler chickens. Humic substances concentrated in CHP but not in MCP could play a crucial role in ileal digestibility, as that has already been described for dry matter digestibility in broilers [7].

The precise effects of humic substances are still under study [16], but two major hypotheses have been proposed: a positive impact on stabilizing the intestine microbiota (amylolytic and cellulolytic bacteria) and an enhancement of digestive enzymatic activities. Previous research indicated that humic acid can increase nutrient digestibility by enhancing villus length [17], which in turn increases the absorption area for nutrients, ultimately improving growth performance [4]. Additionally, humic substances have been found to enhance broiler utilization of Ca [18] and trace elements, as well as to improve feed digestibility by various positive effects on the digestive tract, such as a proper composition of intestinal microbiota, the formation of a protective layer on the intestinal mucosa, and nutrient utilization [4]. Also, the colloidal characteristics of humic substances and their ability to form chelates with different ions have been associated with improved mineral use and enhanced tibia ash and ash retention in poultry [7,19]. These chelating properties, particularly their ability to bind with minerals, mean they could serve as effective Ca sequestering agents. In this way, humic substances substantially reduce the occurrence of insoluble mineral complexes, particularly those formed through interactions between Ca and P.

## 5. Conclusions

The initial objective of this study was to evaluate the impact of phytase and P source on performance parameters and ileal digestibility in broilers. The results indicated that the inclusion of higher doses of phytase in the grower diet improved the FCR of the birds. However, no differences in growth performance were detected between the two phosphate sources under test. At 21 d of age, the use of CHP as the sole source of phosphate in the diet improved P digestibility. In fact, the inclusion of CHP in the diet improved the digestibility of P, DM, OM, and ash, with effects that were generally more pronounced at lower doses of phytase.

The results obtained in the current experiment indicate that the inclusion of CHP in the substitution of MCP as the sole phosphate source in the grower diet might be used to improve P and OM digestibility in broilers, especially with phytase supplementation.

## Figures and Tables

**Table 1:** Ingredient composition and calculated chemical analysis of the experimental diets (grower phase: from 11 to 21 days of age).

Source of Phosphate	MCP ^1^	CHP ^1^
**Ingredient (%)**		
Corn	62.9	62.8
Soybean meal	31.8	31.9
Soy oil	2.24	2.27
Limestone	1.20	1.23
Monocalcium phosphate	0.53	-
HumIPHORA	-	0.55
Sodium chloride	0.35	0.35
DL-Methionine	0.28	0.28
Premix ^2^	0.40	0.40
L-lysine HCl	0.21	0.21
L-threonine	0.08	0.08
L-valine	0.02	0.02
**Determined analysis (%)**		
Moisture	11.9	11.5
Ash	4.92	5.07
Crude protein	19.7	19.9
Ether extract	4.53	4.77
Crude fiber	2.63	2.67
Calcium	0.72	0.72
Phosphorus	0.51	0.52
**Calculated analysis (%)**		
AMEn (kcal/kg)	3030	3030
**Digestible amino acids**		
Lys	1.08	1.08
Met	0.56	0.56
Met+Cys	0.83	0.83
Thr	0.72	0.72
Trp	0.20	0.20
Ile	0.74	0.74
Val	0.86	0.86

^1^ Supplemented with 0, 1000, or 2000 FTU Axtra PHY per kg of feed. ^2^ Included per kilogram of feed: vitamin A, 13,600 IU; vitamin D3, 2720 IU; vitamin E (d alfa tocopherol), 27 mg; vitamin B1, 0.7 mg, vitamin B2, 5.6 mg; vitamin B6, 6.7 mg; vitamin B12, 20 μg; Zn, 52 mg; Cu, 6.8 mg; I, 1.1 mg; Se, 0.14 mg; Fe, 33 mg; niacin, 20 mg; pantothenic acid, 13.2 mg; folic acid, 0.7 mg; biotin, 68 μg; choline chloride.

**Table 2:** Effects of the phosphate (P) source (MCP and CHP) and phytase activity (0, 1000, 2000 FTU/kg) on broiler weight (g) and growth performance (ADG, ADFI, FCR) from 1 to 21 d of age. *n*= 30, 20, and 10 for the P source, phytase activity, and their interaction, respectively.

	Main Effects					Interaction								
	P Source		Phytase Activity (FTU/kg)			MCP			CHP			*p*-Value		
	MCP	CHP	0	1000	2000	0 FTU/kg	1000 FTU/kg	2000 FTU/kg	0 FTU/kg	1000 FTU/kg	2000 FTU/kg	P Source	Phytase	Interaction
0 days	45.4	45.6	45.3	45.8	45.5	45.2	45.7	45.4	45.3	45.9	45.6	0.383	0.256	0.944
10 days	313	312	315	310	313	317	307	317	314	314	309	0.747	0.487	0.211
21 days	1051	1047	1045	1049	1054	1049	1048	1057	1040	1050	1051	0.614	0.596	0.836
**0 to 10 d**														
ADG, g/d	26.8	26.7	27.0	26.4	26.7	27.1	26.1	27.2	26.9	26.8	26.3	0.691	0.398	0.193
ADFI, g/d	29.7	29.7	29.8	29.5	29.8	29.9	29.2	30.0	29.7	29.8	29.5	0.958	0.646	0.295
FCR, g/g	1.109	1.114	1.105	1.116	1.114	1.104	1.118	1.105	1.106	1.114	1.124	0.458	0.411	0.391
**11 to 21 d**														
ADG, g/d	67.1	66.8	66.3	67.2	67.4	66.6	67.4	67.3	66.0	67.0	67.5	0.691	0.333	0.870
ADFI, g/d	89.0	88.0	89.0	88.1	88.5	89.4	88.6	89.1	88.7	87.5	87.8	0.223	0.648	0.963
FCR, g/g	1.327	1.317	1.343 **^b^**	1.311 **^a^**	1.313 **^a^**	1.343	1.315	1.324	1.343	1.307	1.303	0.218	**0.003**	0.539
**0 to 21 d**														
ADG, g/d	47.9	47.7	47.6	47.8	48.0	47.8	47.7	48.2	47.4	47.8	47.9	0.593	0.608	0.839
ADFI, g/d	60.8	60.2	60.8	60.2	60.5	61.1	60.3	60.9	60.6	60.0	60.1	0.258	0.540	0.895
FCR, g/g	1.269	1.263	1.278 **^b^**	1.259 **^a^**	1.259 **^a^**	1.278	1.264	1.264	1.278	1.255	1.255	0.325	**0.016**	0.747

^a,b^ Significant effects are highlighted between treatments using letters a, b (*p* < 0.05).

**Table 3:** Effects of the phosphate source and phytase activity on ileal digestibility (%) of nutrients in broilers at 21 d of age. *n* = 30, 20, and 10 for the phosphate source, phytase activity, and their interaction, respectively.

	Main Effects					Interaction								
	P Source		Phytase Activity (FTU/kg)			MCP			CHP			*p*-Value		
	MCP	CHP	0	1000	2000	0 FTU/kg	1000 FTU/kg	2000 FTU/kg	0 FTU/kg	1000 FTU/kg	2000 FTU/kg	P Source	Phytase	Interaction
Dry matter	61.8	65.3	64.4 **^a^**	64.2 **^a^**	61.9 **^b^**	62.2 **^b^**	61.1 **^b’^**	61.9	66.5 **^a^**	67.4 **^a’^**	61.9	**<** **0** **.001**	**<** **0** **.001**	**<** **0** **.001**
Organic matter	63.3	66.8	66.0 **^a^**	65.8 **^a^**	63.4 **^b^**	63.9 **^b^**	62.6 **^b^**	63.4 **^b^**	68.1 **^a^**	69.0 **^a^**	63.3 **^b^**	**<** **0** **.001**	**<** **0** **.001**	**<** **0** **.001**
Calcium	49.7	51.0	52.5	50.0	48.6	50.9	51.0	47.4	54.1	49.1	49.8	0.334	0.052	0.234
P	60.3 **^b^**	64.0 **^a^**	49.7 **^c^**	65.8 **^b^**	70.9 **^a^**	46.8	64.9	69.1	52.5	66.7	72.7	**<** **0** **.001**	**<** **0** **.001**	0.271
Ash	34.4	37.6	34.7	36.9	36.3	31.8 **^b^**	34.9 **^ab^**	36.4 **^ab^**	37.7 **^a^**	38.9 **^a^**	36.2 **^ab^**	**0.010**	0.186	**0.038**

^a,b,c,a’,b’^ Significant effects are highlighted between treatments using letters a, b, c (*p* < 0.05).

## Data Availability

The original contributions presented in this study are included in the article. Further inquiries can be directed to the corresponding author.

## Abbreviations

The following abbreviations are used in this manuscript:

P	Phosphorus
Ca	Calcium
MCP	Monocalcium Phosphate
CHP	Calcium Humophosphate
FTU	Phytase Activity Expressed in Units
ADG	Average Daily Gain
ADFI	Average Daily Feed Intake
FCR	Feed Conversion Ratio
DM	Dry Matter
OM	Organic Matter
Ti	Titanium
